# The Committee Stage of the National Insurance Bill

**Published:** 1911-07-15

**Authors:** 


### HOSPITALS AND STATE INSURANCE.

### THE COMMITTEE STAGE OF THE NATIONAL INSURANCE BILL.

At the time of writing seven clauses of the National Insurance Bill had been considered, and clause 8 was under discussion. In view of the fact that the measure contains eighty-seven clauses and nine schedules, it is evident that the Committee will have to work at high pressure if the Bill is to be passed this session. That the Chancellor of the Exchequer is anxious to satisfy to some extent the demands of medical practitioners became evident at the beginning of the Committee stage, for during the consideration of the first clause he accepted an amendment, moved by Mr. Joynson Hicks, providing that no person with an income of over £160 a year should become a voluntary contributor. The acceptance of this amendment means that the larger part of the scheme so far as it affects voluntary contributors is dropped. From the point of view of medical practitioners the concession is therefore extremely important. Under the Bill as introduced there was no income limit for the voluntary contributor, and, as was pointed out in The Hospital of May 13, 1911 (page 167), this was tantamount to a proposal for the nationalisation of medical service except in the case of the upper, upper-middle, and wealthy classes. In accepting the principle of limiting the income of voluntary contributors, the Chancellor of the Exchequer said one of the greatest difficulties with medical practitioners was their apprehension that the class of patients whose payment at present enabled them to deal liberally with the poorer members of the community would be put on contract terms. The question of reducing the income limit of compulsory contributors has yet to be discussed. During the second day's proceedings there was a long debate on Clause 2, which provides that certain persons shall be entitled to a certificate exempting them from liability to become insured under the Act. In the course of the debate the Chancellor said he was considering whether in the case of certain classes, including hospital nurses, it was possible to deduct from their contributions an actuarial equivalent of the cost of what they were getting under their service, and simply charge what is enough to provide for permanent disability. The clauses which mainly affect the position of medical practitioners and hospitals have not yet been reached, but questions affecting medical practice are constantly cropping up in the debates. For instance, during the discussion of an amendment of Clause 4, which provides for rates and rules for contributions by employed contributors and their employers, Mr. Lloyd George made an interesting statement regarding the incidence of sickness. It appears to be a paradox, but during the last few years insurance offices have profited by the improved health of the community, whilst sickness societies have lost by it.

When clause 8 was reached on Tuesday night there was an hour's debate on a motion by Mr. Joynson Hicks that sub-section A should be omitted. This sub-section describes the character of one of the benefits conferred under the Bill as "Medical treatment and attendance, including the provision of proper and sufficient medicines (in this Act called 'medical benefit')," and his object in moving the amendment was not to leave out the benefits, but to give the Chancellor an opportunity of informing the Committee of his intentions regarding the medical profession. He submitted that the House could not vote for or against medical benefits unless they knew what they were and how they were going to be given. In the course of the debate Dr. Addison expressed the hope that the Chancellor would resist this amendmnt; he was confident that to engage in any detailed discussion at this stage would do much harm and no good. The Chancellor said it would be preferable to raise the whole question on clause 14, and he was confident that by the time this clause was reached an arrangement would have been arrived at which would be very satisfactory to the medical profession. After some further discussion, and on the Chancellor undertaking to recommit the Bill in respect of clause 8, the amendment was by leave withdrawn. This preliminary skirmish seems to indicate that when clause 14 is reached medical practitioners will find many supporters in the impending fight.

The discussion on the Financial Resolution relating to the Bill contained frequent references to the question of the treatment of phthisis. The Chancellor expressed the hope that the million and a-half pounds sterling that had been set aside for sanatoria would not be spent upon expensive buildings. He hoped to have the advantage of the advice of eminent medical men on the Advisory Committee who would be able to advise from time to time as to the best way of expending the money. The present idea was to spend the million pounds a year, to be raised towards maintaining the sanatoria, on treatment on the open-air principle, and that other funds should be used for the purpose of treating patients in their own homes. Further questions relating to the medical and sanatorium benefits under the proposed scheme have been asked and answered. Sir John Rolleston asked whether the Chancellor's attention had been called to the fact that during the ten years ending 1908 the death-rate from pulmonary tuberculosis was 13.3 per 10,000 of the population of the United Kingdom, and 17.9 per 10,000 of the population of that part of the German Empire for which returns have been furnished. In reply, Mr. Lloyd George said it was undoubtedly the case that the rate of mortality from pulmonary tuberculosis is higher in Germany than in the United Kingdom. There has, however, been a large reduction in the death-rate from this cause in Germany since the sickness and invalidity insurance laws have been in full operation, and German authorities attribute the reduction in part to the beneficial influence of these laws upon the health of the nation, and particularly to the measures adopted—including an extensive system of sanatoria—for the treatment of consumption in the early stages of the disease.

Wednesday's Parliamentary papers contain a number of amendments handed in by the Chancellor of the Exchequer. The proposed amendments of clause 14 provide that the local Health Committee shall make arrangements with medical practitioners in accordance with regulations made by the Insurance Commissioners, and that these regulations shall provide for a free choice of medical attendant by insurance patients. Another official amendment provides for medical representation on the Advisory Committee and more efficient representation on the Health Committees. Amendments of this character will certainly facilitate the passage of the Bill.

